# Impact of the COVID-19 Pandemic and Lockdown on Anxiety, Depression and Nursing Burden of Caregivers in Alzheimer's Disease, Dementia With Lewy Bodies and Mild Cognitive Impairment in China: A 1-Year Follow-Up Study

**DOI:** 10.3389/fpsyt.2022.921535

**Published:** 2022-07-07

**Authors:** Xinran Bao, Junying Xu, Qingbo Meng, Jinghuan Gan, Xiao-Dan Wang, Hao Wu, Shuai Liu, Yong Ji

**Affiliations:** ^1^Clinical College of Neurology, Neurosurgery and Neurorehabilitation, Tianjin Medical University, Tianjin, China; ^2^Tianjin Key Laboratory of Cerebrovascular and of Neurodegenerative Diseases, Department of Neurology, Tianjin Dementia Institute, Tianjin Huanhu Hospital, Tianjin, China; ^3^Department of Neurology, First Hospital of Qinhuangdao, Hebei, China; ^4^Department of Neurology, Baodi Clinical College of Tianjin Medical University, Tianjin, China; ^5^Department of Neurology, China National Clinical Research Center for Neurological Diseases, Beijing Tiantan Hospital, Capital Medical University, Beijing, China

**Keywords:** COVID-19, social isolation, physical activities, caregiver burden, Dementia with Lewy Bodies, Alzheimer's disease

## Abstract

**Background:**

Many countries have adopted lockdown strategies to prevent the spread of COVID-19. The goal of this study was to investigate the effects of the pandemic on anxiety, depression and care burden in caregivers of nursing patients with Alzheimer's disease (AD), Dementia with Lewy Bodies (DLB) and Mild Cognitive Impairment (MCI), over a one-year period.

**Methods:**

We collected data on consecutive patients and their caregivers recruited at T0 (from 30 September to 31 December 2019) before the pandemic of COVID-19 at the memory clinic of Tianjin Huanhu Hospital. The patients and caregivers were followed up on face-to-face at T1 (from 30 September to 31 December 2020) during the pandemic to assess changes in physical activity, social contact, sleep quality, caregiver burden, anxiety and depression.

**Results:**

A total of 105 AD, 22 DLB and 50 MCI patients and caregivers were enrolled. A total of 36.6 % of the AD, 81.6% of the DLB, 38% of the MCI caregivers had worsening ZBI, whereas 31.7 % of the AD, 54.4% of the DLB, 26 % of the MCI caregivers had worsening GAD-7, and 29.6 % of the AD, 54.4% of the DLB, and 32 % of the MCI caregivers had worsening PHQ-9. DLB caregivers exhibited a rapid deterioration of ZBI (by 4.27 ± 5.43, *P* < 0.001), GAD-7 (by 2.23 ± 3.26, *P* = 0.003) and PHQ-9 (by 1.32 ± 2.25, *P* = 0.003) compared to AD and MCI caregivers.

**Conclusion:**

Social isolation, physical inactivity and sleep disturbance after lockdown for at least 12 months were significantly related to increased caregiver burden and worsened psychological states of caregivers of AD, DLB and MCI sufferers, especially among DLB caregivers.

## Introduction

The outbreak of coronavirus disease 2019 (COVID-19) brought about severe acute respiratory syndrome coronavirus 2 (SARS-CoV-2) and caused millions of deaths worldwide, especially among senior citizens (1), the mortality rate reached 26.2% in elderly individuals and 62.2% in elderly individuals with dementia (2). Numerous countries have imposed a variety of measures, including quarantine, to stop the virus from spreading (3). However, continued mutation of the virus has forced many countries to extend quarantine periods (4).

Under the influence of the COVID-19 pandemic and government policy, events and gatherings were canceled, public places were closed, people were forced to work from home, and physical activity (PA) and social contact were limited to the greatest possible extent (5). PA levels among older adults decreased by 26.5%, as reported during the pandemic (6). Many studies have demonstrated that disrupted living habits, such as reduced PA, social isolation and disturbed sleep due to confinement, have adversely affected the physical and mental status of seniors during the COVID-19 pandemic (7, 8). A systematic review (9) showed the general population, particular older adults, had higher pool prevalence rates of depression, anxiety, psychological distress, poor sleep quality or insomnia during COVID-19 pandemic. Moreover, a previous study has confirmed that the prevalence of anxiety and depressive symptoms in people affected by quarantine was double compared to unaffected individuals (10). Family caregivers, especially those tending individuals suffering from cognition impairment, were more prone to suffering anxiety, depression and heavy nursing burdens (11, 12). There are only limited studies on the effects of COVID-19 quarantine on the psychological status (anxiety, depression) and care burden of caregivers that include follow-up for at least 1 year during the COVID-19 pandemic (13, 14). Such studies have not discussed the differences among such effects according to the cognitive impairment subtypes of the individuals caregivers care for.

Our study aimed at inspecting the change in psychological status and care burden of family caregivers during the COVID-19 pandemic among caregivers who care for individuals suffering from Alzheimer's disease (AD), Dementia with Lewy Bodies (DLB) and patients with Mild Cognitive Impairment (MCI). In addition, we investigated predictive factors, especially in terms of physical activity, social contact and sleep disturbance, with respect to the worsening or improvement of the issues of concern.

## Methods

### Design and Participants

This study was conducted at the memory clinic of Tianjin Huanhu Hospital, Tianjin, China. A total of 177 paired consecutive patients with AD (*n* = 105), DLB (*n* = 22) and MCI (*n* = 50) and their caregivers were recruited between 30 September and 31 December 2019 before the pandemic of COVID-19 (Table 1). The 177 enrolled patients and caregivers (AD *n* = 105, DLB *n* = 22, MCI *n* = 50) were completed follow-up on face-to-face between 30 September and 31 December 2020 one year after during the pandemic (Table 2). Participants (patients and/or caregivers) who failed to visit the memory clinic regularly or whose number of visits was inadequate to enable obtaining sufficient information were excluded from the study. Caregivers who had <8 h contact with their patients per day were not included. Our study was designed to include two observation points: a baseline observation at T0 (from 30 September to 31 December 2019) and a 1-year follow-up observation at T1 (from 30 September to 31 December 2020). The neurologists retrospectively investigated the subjects to evaluate the changes in the information gathered in the initial assessment.

**Table 1:** Baseline characteristics of the AD, DLB and MCI patients and their caregivers.

**Characteristic**	**AD**	**DLB**	**MCI**	***P***
	**(*n =* 105)**	**(*n =* 22)**	**(*n =* 50)**	
**Patients**
Male, *n* (%)	44 (41.9)	12 (54.5)	21 (42)	0.536
Age, years	71.55 ± 8.14	73.96 ± 7.87^d^	68.67 ± 8.59	0.030^*^
Course of disease, year	4.3 ± 2.39^b^	3.02 ± 1.61	2.76 ± 1.62	<0.001^***^
MMSE	13.02 ± 7.02^c^	13.73 ± 7.01^d^	25.30 ± 2.51	<0.001^***^
MoCA	8.83 ± 5.68^c^	9.86 ± 5.89^d^	21.02 ± 2.68	<0.001^***^
NPI	9.56 ± 11.25^c^	18.64 ± 14.36^d^^f^	2.70 ± 4.99	<0.001^***^
**Caregivers**				
Female, *n* (%)	40 (49.4)	12 (54.5)	26 (59.1)	0.576
Age, years (Mean±SD)	65.31 ± 10.35^a^	57.43 ± 11.04^e^	58.72 ± 11.90	0.001^**^
**Relationship with patient**				
Spouse, *n* (%)	65 (61.9)	14 (63.64)	23 (46)	0.052
Child, *n* (%)	14 (13.33)	8 (36.36)	17 (34)	
Maids, *n* (%)	3 (2.86)	0 (0)	1 (2)	
Others, *n* (%)	23 (21.9)	0 (0)	9 (18)	
Living with patient, Yes	77 (73.3)	19 (86.4)	34 (68)	0.177
**Marital status**, ***n*** **(%)**				0.584
Unmarried	2 (2)	0 (0)	2 (4)	
Married	102 (97)	22 (100)	47 (94)	
Divorced	0 (0)	0 (0)	1 (2)	
Unknown	1 (1)	0 (0)	0 (0)	
**Education attainment**, ***n*** **(%)**				0.286
Illiterate	2 (1.9)	0 (0)	1 (2)	
Primary school	10 (9.5)	2 (9.1)	2 (4)	
Middle school	24 (22.9)	6 (27.3)	9 (18)	
High school	17 (16.2)	8 (36.4)	11 (22)	
College	24 (22.9)	5 (22.8)	11 (22)	
Missed	28 (26.7)	1 (4.6)	9 (18)	
Years of caring	5.01 ± 12.13	7.22 ± 13.63	5.58 ± 12.31	0.791

*MCI vs. AD vs. DLB:*

* *P < 0.05,*

** *P < 0.01,*

*** *P < 0.001*.

*MCI vs. AD:*

a *P < 0.05,*

b *P < 0.01,*

c *P < 0.001*.

*MCI vs. DLB:*

d *P < 0.001*.

*AD vs. DLB:*

e *P < 0.01,*

f *P < 0.001*.

*AD, Alzheimer's disease; DLB, Dementia with Lewy bodies; MCI, Mild Cognitive Impairment; MMSE, Mini-Mental State Examination; MoCA, Montreal Cognitive Assessment; NPI, Neuropsychiatric Inventory*.

**Table 2:** Comparison of baseline (T0) and 1-year follow-up (T1) data for caregivers of individuals afflicted with AD, DLB and MCI.

	**AD (*****n =*** **105)**				**DLB (*****n =*** **22)**				**MCI (*****n =*** **50)**			
	**Baseline**	**Follow-up**	**Change**	***P***	**Baseline**	**Follow-up**	**Change**	***P***	**Baseline**	**Follow-up**	**Change**	***P***
MMSE	13.02 ± 7.02	11.43 ± 7.40	1.59 ± 3.35^a^	<0.001^***^	13.73 ± 7.01	10.09 ± 6.91	3.64 ± 5.49^d^	0.005^**^	25.30 ± 2.51	23.98 ± 5.79	1.32 ± 5.22	0.080
MoCA	8.83 ± 5.68	7.80 ± 5.87	1.03 ± 3.24	0.002^**^	9.86 ± 5.89	7.32 ± 5.37	2.55 ± 4.98	0.026^*^	21.02 ± 2.68	20.14 ± 5.51	0.88 ± 4.33	0.157
NPI	9.56 ± 11.26	12.27 ± 13.84	2.70 ± 8.25^g^	0.001^**^	18.64 ± 14.36	20.91 ± 14.75	2.27 ± 10.49	0.321	2.70 ± 4.99	2.84 ± 5.23	0.14 ± 2.65	0.710
ZBI	19.69 ± 22.33	21.40 ± 24.067	1.48 ± 5.20^i^	<0.001^***^	12.45 ± 12.10	16.73 ± 16.46	4.27 ± 5.43^c^	<0.001^***^	16 ± 15.19	17.25 ± 16.17	1.61 ± 4.71^f^	0.032^*^
PHQ-9	2.50 ± 4.87	2.52 ± 4.81	0.05 ± 0.93^g^	0.006^**^	1.14 ± 1.28	2.45 ± 2.20	1.32 ± 2.25^b^	0.003^**^	2.11 ± 2.96	2.61 ± 3.33	0.50 ± 1.29^d^	0.007^**^
GAD-7	2.44 ± 4.73	2.67 ± 4.54	0.27 ± 1.94^h^	0.002^**^	1.14 ± 1.32	3.36 ± 3.27	2.23 ± 3.26^b^	0.003^**^	1.91 ± 3.72	2.70 ± 4.47	0.80 ± 2.70^e^	0.016^**^
PSQI	18.05 ± 5.01	17.67 ± 5.82	0.05 ± 0.75	0.032^*^	16.14 ± 2.48	17.32 ± 3.66	1.18 ± 1.87^a^	0.008^**^	19.04 ± 5.44	19.8 ± 4.85	0.32 ± 1.64	0.119
Social contact	8.60 ± 2.70	7.72 ± 2.91	−0.89 ± 2.00^i^	<0.001^***^	8.45 ± 3.39	6.91 ± 2.98	−1.55 ± 3.14	0.024^*^	7.59 ± 3.11	6.89 ± 3.15	−0.70 ± 2.25^f^	0.005^**^
PA	4.31 ± 3.42	3.04 ± 2.59	−1.27 ± 2.26	<0.001^***^	1.57 ± 2.60	0.30 ± 0.81	−1.27 ± 2.62	0.026^*^	3.44 ± 3.32	2.07 ± 2.39	−1.37 ± 2.57	<0.001^***^
Caring time (h)	19.52 ± 8.35	20.22 ± 7.76	0.70 ± 3.20^i^	<0.001^***^	17.59 ± 8.30	20.45 ± 6.17	2.86 ± 6.31	0.066	18.45 ± 7.50	19.63 ± 6.73	1.32 ± 4.09^f^	0.008^**^

*Baseline mean compared with 1-year follow-up:*

* *P < 0.05,*

** *P < 0.01,*

*** *P < 0.001*.

*Changes in the evaluated scores between AD and DLB:*

a *P < 0.05,*

b *P < 0.01,*

c *P < 0.001*.

*Changes in the evaluated scores between DLB and MCI:*

d *P < 0.05,*

e *P < 0.01,*

f *P < 0.001.*

*Changes in the evaluated scores between MCI and AD:*

g *P < 0.05,*

h *P < 0.01,*

i *P < 0.001*.

*AD, Alzheimer's disease; DLB, Dementia with Lewy bodies; MCI, Mild Cognitive Impairment; MMSE, Mini-Mental State Examination; MoCA, Montreal Cognitive Assessment; NPI, Neuropsychiatric Inventory; ZBI, Zarit Burden Interview; PHQ-9, Patient Health Questionnaire; GAD-7, Generalized Anxiety Disorder Scale; PSQI, Pittsburgh Sleep Quality Index; PA, Physical activity*.

### Measures

#### Patient Measurement

All patients underwent extensive neurological, neuropsychological, laboratory and neuroimaging assessments to ensure that they met all dementia criteria. The diagnoses of AD and MCI were based on the National Institute on Aging and Alzheimer's Association (NIA-AA) criteria (15), and the diagnosis of DLB was made according to the fourth consensus report of the DLB consortium (16). Blood tests, neurological examination, neuroimaging (including CT scans or MRI), and positron emission computerized tomography (including FDG-PET and amyloid PET) were performed to confirm the diagnosis if necessary. All clinical diagnoses of dementia were made by at least two expert neurologists in agreement. Severe vision or acoustic loss, physical handicap, lack of follow-up, recent onset delirium, stroke, and life-threatening conditions were all considered exclusion criteria. At the 1-year follow-up, we reassessed the neurological examination and rectified the diagnosis, excluding participants in the MCI group who developed dementia or rectified the diagnosis as other diseases in the DLB and AD groups.

Age, gender, cognitive function, neuropsychiatric symptoms (NPS), and disease duration were collected and assessed. Cognitive function was assessed using the Mini-Mental State Examination (MMSE) (17) and Montreal Cognitive Assessment (MoCA) (18). The Neuropsychiatric Inventory (NPI) was used to assess the frequency and degree of NPS (19).

The tests administered for patients were MMSE, MoCA and NPI at T0 and T1.

#### Caregiver Measurement

Demographic-situational data included age, gender, relation with the patient, marital status, hours/day caring the patient, physical activity, social contact, caregiver burden, depression, anxiety and sleep quality.

A semi-structured health-related questionnaire survey (20) containing questions on regularity, duration, and intensity was used to assess physical activity. Social contact was evaluated by using a semi-structured questionnaire adapted by a cohort study that assessed the quantity and frequency of interaction with relatives and friends (21). The care burden of the caregivers was estimated using the Zarit Burden Interview (ZBI) (22). The ZBI consists of 22 items rated on a five-point scale ranging from 0 (none) to 4 (overburdened). The higher the score, the greater the burden. Anxiety was assessed using the Generalized Anxiety Disorder Scale (GAD-7) (23) is a 7-item scale (range 0–21). The higher the score, the greater the psychological pressure present. Depression was evaluated with the Patient Health Questionnaire (PHQ-9) (24), which includes nine items, scored by frequency: not at all (0), several days (1), more than half the days (2) and almost every day (3). Higher scores on the PHQ-9 indicate greater depression. The Pittsburgh Sleep Quality Index (PSQI) was applied to assess subjective sleep quality, the scale ranks sleep on a scale from 0–21, whereby a higher global PSQI score indicates poorer sleep quality (25).

The tests administered for caregivers were ZBI, GAD-7, PHQ-9, PSQI, social contact, physical activity (PA) and caring time at T0 and T1.

#### Statistical Analysis

Clinical characteristics, recognition status, NPI, GAD-7, PHQ-9, and PSQI are presented as means ± standard deviation for continuous variables and numbers (percentages) for categorical variables in the three groups of gender, age, disease duration, education level and marital status. The Shapiro-Wilk test and Kolmogorov-Smirnov test were used to analyze the normality of the distribution. Among the AD, DLB and MCI groups, the differences in descriptive statistics that were normally distributed were analyzed using analysis of variance (ANOVA), and the statistics which were nonnormally distributed were tested using Kruskal-Wallis test. Finally, the categorical variables were tested by using the Chi-squared test, Pearson's test and Fisher's exact test. The comparison of data at baseline (T0) and 1-year follow-up (T1) was performed by Wilcoxon test if the statistics were nonnormally distributed and the comparison was performed by pared-samples T test if the statistics were normally distributed. Correlation analysis was conducted by using partial correlation adjusted for age and gender. Multiple linear regression analysis was used to identify possible risk factors for worsening GAD-7, PHQ-9 and ZBI scores in caregivers of AD and DLB patients during the COVID-19 pandemic. All statistics were analyzed using SPSS Statistics 25.0. A *P*-value <0.05 was regarded as statistically significant.

## Results

A total of 177 patients and their corresponding caregivers (105 AD, 22 DLB, 50 MCI) completed the 1-year longitudinal follow-up survey.

### Baseline Patient and Caregiver Characteristics in the Three Groups

The baseline attributes of the patients are shown in Table 1. The age of the patients with DLB was significantly higher than that of the MCI group (*P* = 0.030) but not significantly different from that of those with AD. The course of disease was much higher in the AD group (*P* < 0.001) than in the MCI but not significantly different from that of those with DLB. The gender of the patients showed no statistical significance in the AD, DLB and MCI groups. The MMSE and MoCA scores were lower in the AD and DLB groups than in the MCI group (*P* < 0.001), however, they were similar in the AD and DLB groups. Patients afflicted with DLB demonstrated higher NPI marks than those afflicted with AD and MCI (*P* < 0.001), and the difference was significant.

The demographic characteristics of the caregivers in the three subtypes are shown in Table 1. These subtypes were well paired for caregiver education attainment, whether they lived with their patients, the relationships with their patients and years of caring. Approximately fifty percent of the caregivers were spouses. More than fifty percent of the caregivers from the three groups were female. More than fifty percent of all caregivers in the AD (62.0%), DLB (86.5%) and MCI (62.0%) groups had finished middle school. The age of the caregivers in the AD group was distinctly higher than that of those in the DLB and MCI groups (*P* = 0.001), while age in the latter groups showed no significant differences.

### Changes in the Three Groups Between Baseline and 1-Year Follow-Up

**Figure 1:**
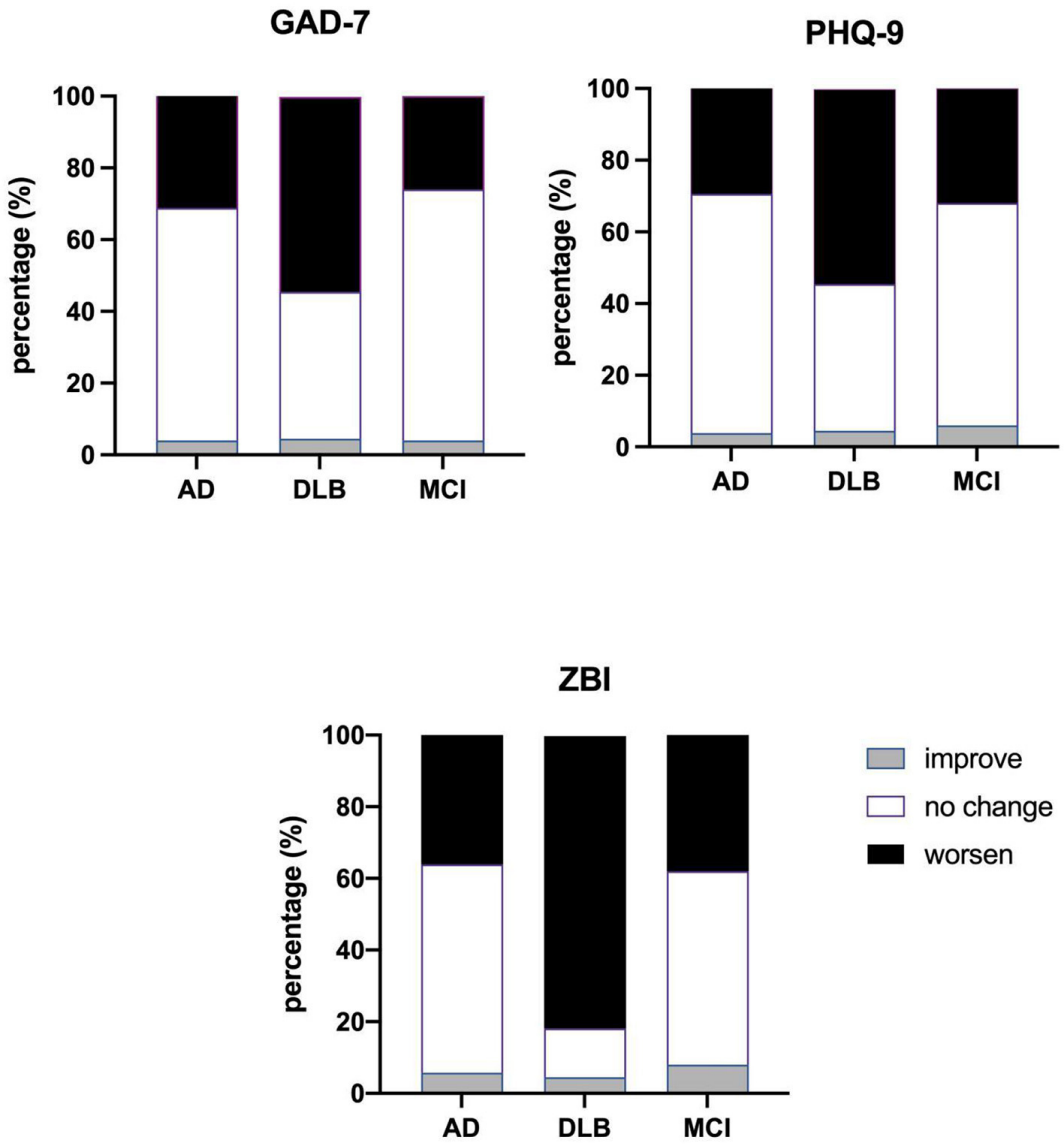
Comparison of changes in GAD-7, PHQ-9 and ZBI in the three groups. AD, Alzheimer's disease; DLB, Dementia with Lewy bodies; MCI, Mild Cognitive Impairment; ZBI, Zarit Burden Interview; GAD-7, Generalized Anxiety Disorder Scale; PHQ-9, Patient Health Questionnaire.

As shown in Figure 1, all of the GAD-7, PHQ-9 and ZBI scores of the caregivers increased in the three groups during the 2019 coronavirus epidemic period, and the changes in GAD-7, PHQ-9 and ZBI were prominent in the DLB group compared with the AD and MCI groups. More than half of the caregivers in the DLB group exhibited worsening GAD-7, PHQ-9 and ZBI.

As shown in Table 2, the scores of MMSE and MoCA in the AD group decreased significantly at T1 (by 1.59 ± 3.35, *P* < 0.001, 1.03 ± 3.24, *P* = 0.002) compared to T0, similar results were observed in the DLB group (by 3.64 ± 5.49, *P* = 0.005, 2.55 ± 4.98, *P* = 0.026). While MMSE (by 1.32 ± 5.22, *P* = 0.080) and MoCA (by 0.88 ± 4.33, *P* = 0.157) showed no significant change in the MCI group. There was a significant decrease in physical activity in caregivers tending AD (by 1.27 ± 2.26, *P* < 0.001), DLB (by 1.27 ± 2.62, *P* = 0.026) and MCI (by 1.37 ± 2.57, *P* < 0.001) sufferers at T1 compared to T0, and physical activity declined similarly in all three groups (AD vs. DLB *P* = 0.635, DLB vs. MCI *P* = 0.969, AD vs. MCI *P* = 0.809). Social contact declined more obviously in the AD (by 0.89 ± 2.00, *P* < 0.001) and DLB (by 1.55 ± 3.14, *P* = 0.024) groups. There was a significant decrease in PSQI in caregivers tending AD (by 0.05 ± 0.75, *P* = 0.032), DLB (by 1.18 ± 1.87, *P* = 0.008) and MCI (by 0.32 ± 1.64, *P* = 0.119) sufferers. Psychological status, evaluated by the PHQ-9 and GAD-7, and care burden, evaluated by the ZBI score, increased significantly in all three groups, while the PHQ-9 (by 1.32 ± 2.25, *P* = 0.003), GAD-7 (by 2.23 ± 3.26, *P* = 0.003) and ZBI scores (by 4.27 ± 5.43, *P* < 0.001) increased the most prominently and significantly in DLB caregivers at T1.

### Predictors of Anxiety, Depression and Care Burden in the AD and DLB Caregivers

We examined the data for the associations between deterioration of mental state (GAD-7, PHQ-9), increased care burden (ZBI) and presumed risk factors, including baseline MMSE score, NPI change, social activity change, physical activity change, caring time change, PSQI change, and patients' psychological status (GAD-7, PHQ-9) in caregivers of patients afflicted with DLB and AD (Table 3). MMSE and MoCA at baseline were not related to caregivers' anxiety, depression and care burden change. We discovered that declines in social activity (*r* = 0.378, *P* < 0.001) and physical activity (*r* = 0.316, *P* = 0.001) were positively correlated with worsened GAD-7 in caregivers of AD patients, and declines in social activity (*r* = 0.761, *P* < 0.001) and physical activity (*r* = 0.600, *P* = 0.007) were also positively correlated with worsened GAD-7 in caregivers of DLB patients. Social activity decline (*r* = 0.355, *P* < 0.001) was positively correlated with the decline of PHQ-9 scores in caregivers of AD patients. The worsening of the GAD-7 (*r* = 0.456, *P* < 0.001) and PHQ-9 (*r* = 0.394, *P* < 0.001) scores was positively related to the increased care burden (ZBI) in the AD subtype. Worsened GAD-7 (*r* = 0.464, *P* = 0.045) and declining physical activity (*r* = 0.728, *P* < 0.001) were positively correlated with the care burden (ZBI) of DLB caregivers. Physical activity, social contact and sleep disturbance were not correlated with PHQ-9 scores in caregivers of patients with DLB. Other factors were also correlated with GAD-7 and PHQ-9 in the AD subtype, including sleep disturbance (*r* = 0.464, *P* < 0.001 and *r* = 0.315, *P* = 0.001, respectively). Caring time, baseline MMSE of patients and NPI change and relationships to patients were not significantly correlated with the GAD-7, PHQ-9, and ZBI of the AD and DLB subtypes.

**Table 3:** Correlation of worsened GAD-7, PHQ-9 and ZBI with hypothesized related factors in the AD and DLB caregivers.

	**AD (*****n =*** **105)**						**DLB (*****n =*** **22)**					
	**GAD-7 worse**		**PHQ-9 worse**		**ZBI worse**		**GAD-7 worse**		**PHQ-9 worse**		**ZBI worse**	
	**r**	***P***	**r**	***P***	**r**	***P***	**r**	***P***	**r**	***P***	**r**	***P***
**Patients**												
MMSE (baseline)	−0.033	0.739	0.012	0.901	0.021	0.834	−0.034	0.891	0.094	0.702	0.055	0.823
MoCA(baseline)	−0.055	0.654	−0.033	0.788	0.026	0.830	−0.157	0.520	0.012	0.961	0.008	0.973
NPI (change)	−0.031	0.751	−0.06	0.544	−0.079	0.425	−0.246	0.310	−0.076	0.757	−0.091	0.710
**Caregivers**												
Social activity (change)	−0.378	< 0.001^***^	−0.355	< 0.001^***^	−0.183	0.062	−0.761	<0.001^***^	−0.192	0.430	−0.220	0.366
PA (change)	−0.316	0.001^**^	−0.169	0.085	−0.235	0.016^**^	−0.600	0.007^**^	−0.171	0.484	−0.728	<0.001^***^
Caring time (change)	0.127	0.196	0.054	0.581	0.042	0.673	0.534	0.019^*^	−0.236	0.330	0.020	0.936
PSQI (change)	0.464	< 0.001^***^	0.315	0.001^**^	0.205	0.036^*^	0.251	0.300	0.389	0.100	0.437	0.062
GAD-7 (change)	–	–	0.725	< 0.001^***^	0.456	< 0.001^***^	–	–	0.310	0.197	0.464	0.045
PHQ-9 (change)	0.725	< 0.001^***^	–	–	0.394	< 0.001^***^	0.31	0.197	–	–	0.239	0.325

*AD, Alzheimer's disease; DLB, Dementia with Lewy bodies; MMSE, Mini-Mental State Examination; MoCA, Montreal Cognitive Assessment; NPI, Neuropsychiatric Inventory; ZBI, Zarit Burden Interview; PHQ-9, Patient Health Questionnaire; GAD-7, Generalized Anxiety Disorder Scale; PSQI, Pittsburgh Sleep Quality Index*.

* *P < 0.05,*

** *P < 0.01,*

*** *P < 0.001; adjusted for age and gender*.

Multiple linear regression analysis was performed to select factors predictive of increased care burden (ZBI) and worsening mental status (GAD-7 and PHQ-9) in AD and DLB caregivers (Table 4). It was found that lower PA and sleep quality predicted a more rapid worsening of anxiety in AD caregivers. A decline in sleep disturbance and social contact among caregivers can predict the depression status of an AD caregiver. The GAD-7 score can predict an increased burden of AD caregivers. In DLB caregivers, the decline in PA and social contact can predict increased anxiety, and the decline in PA can predict increased burden, but neither of PA, social activity or sleep disturbance can predict the depression status of caregivers.

**Table 4:** Multiple linear regression analysis of GAD-7, PHQ-9, and ZBI scores in the caregivers of AD and DLB patients.

	**ZBI**		**GAD-7**		**PHQ-9**	
	**Beta (SD)**	***P***	**Beta (SD)**	***P***	**Beta (SD)**	***P***
**AD (*****n =*** **105)**						
Physical activity change	−0.128	0.223	−0.214	0.019^*^	−0.011	0.918
Social contact change	0.023	0.825	−0.182	0.053	−0.267	0.011^*^
PSQI change	−0.019	0.862	0.396	<0.001^***^	0.263	<0.001^***^
Caring time	−0.063	0.540			0.091	0.373
GAD-7 increase	0.441	<0.001^***^				
Dubin-Watson		1.973		1.566		1.585
Adjusted *R*^2^		0.181		0.300		0.152
*P*-value of *F*		0.001		0.001		0.001
**DLB (*****n =*** **22)**						
Physical activity change	−0.780	<0.001^***^	−0.326	<0.001^***^	−0.354	0.225
Social contact change	−0.014	0.934	−0.503	0.002^**^	−0.269	0.277
PSQI change	0.183	0.292	0.153	0.300	0.173	0.484
Caring time	−0.221	0.245	0.255	0.122	−0.235	0.415
Dubin-Watson		1.522		2.392		2.039
Adjusted R^2^		0.569		0.689		0.354
*P*-value of *F*		0.001		0.000		0.036

*AD, Alzheimer's disease; DLB, Dementia with Lewy bodies; ZBI, Zarit Burden Interview; PHQ-9, Patient Health Questionnaire; GAD-7, Generalized Anxiety Disorder Scale; PSQI, Pittsburgh Sleep Quality Index.*

* *P < 0.05,*

** *P < 0.01,*

*** *P < 0.001*.

## Discussion

This study is the first longitudinal 1-year follow-up study on caregivers of those afflicted with cognition impairment during the COVID-19 pandemic. The study focused on caregivers of AD, DLB and MCI sufferers who experienced quarantine for approximately 12 months with their patients during the pandemic. We aimed to investigate the impact of quarantine on the worsening of depression and anxiety status and the change in the nursing burden of the caregivers of AD, DLB and MCI sufferers. We found that the anxiety, depression and care burden of all subtypes increased. In addition, a sudden decline in PA and social contact during isolation predicted an increase in GAD-7 and PHQ-9 scores in AD caregivers after 1 year of follow-up, and the increased GAD-7 scores indirectly led to an increase in care burden in such caregivers. Sudden declines in PA and social contact during quarantine were predictive factors of an increase in GAD-7 score in caregivers of DLB sufferers, and PA alone was a predictive factor of an increase in ZBI in DLB caregivers.

Our longitudinal study demonstrated different extents of deterioration of depression, anxiety, care burden during COVID-19 quarantine in AD, DLB and MCI caregivers. At the 1-year follow-up, we found that AD caregivers had an average anxiety degree (GAD-7) increase of 0.27, a depression degree (PHQ-9) of 0.05 and a care burden (ZBI) of 1.48. At the 1-year follow-up, we also found that caregivers in the DLB group had an average anxiety degree (GAD-7) increase of 2.23, a depression degree (PHQ-9) of 1.32 and a care burden (ZBI) of 4.27, which were the highest among the 3 groups and in accordance with a study by Svendsboe (26). These findings might be attributable to the different clinical profiles of DLB and AD patients. Patients with DLB generally have more severe psychiatric problems and a higher probability of sleep disorders than those with AD, consequently DLB caregivers experienced the highest caring time surge over the 1-year observation period. According to our statistics data (Table 2), the magnitude of change in caring time was the greatest for DLB caregivers, but it was not statistically significant. And this may due to the limitations of our sample size. As a result, DLB caregivers exhibited the largest decline in PA, social activity and sleep quality in our study.

The analyzed evidence supports the role of quarantine in the increase in anxiety, deterioration of depression status and exacerbation of care burden of those providing care to individuals with cognitive impairment. Furthermore, according to multiple linear regression, we found that the decrease in social contact was related to the increase in the GAD-7 scores in caregivers tending AD and DLB sufferers, while the decrease in social contact was related to the increase in PHQ-9 scores in the caregivers of AD sufferers during the COVID-19 pandemic. Many studies argue that social contact relieves anxiety and related disorders by activating a neural reward system, regulating the HPA axis, and regulating and secreting neurotransmitters, including oxytocin and opioids (27). We found that the decline in PA was related to the increase in the GAD-7 scores in caregivers of AD sufferers and the GAD-7 and ZBI scores of caregivers of DLB sufferers during the observation period. Studies also suggest that physical activity may promote mental health by decreasing anxiety and depression symptoms through downregulating TNF-α (28, 29). A study in Japan underscores the importance of main PA and social activity during the COVID-19 pandemic (30).

Given that previous studies were performed immediately after 1 to 2 months of confinement (31), the differences between previous studies and our study may be due to the impact of short-term vs. long-term confinement on family caregivers. The literature on this topic is scarce. Patients and caregivers easily developed acute stress disorder immediately after isolation which refers to the development of transient emotional as a result of exposure to an event or situation (32). These symptoms are usually short-lived and will diminish within months. We considered 12 months were sufficient for an acute stress disorder in patients and their corresponding caregivers to be alleviated as they became accommodated to their new lifestyle (33). According to one previous study, patients and caregivers are prone to experience worse conditions immediately after quarantine than 12 months later (34).

As we investigated in a previous study, the joint harm of anxiety and depression is strongly related to caregiver burden by directly affecting caregiver physical and mental conditions and satisfaction with the present situation and thus increased caregiver burden. This finding is in accordance with another study (35). PA was correlated with and could predict anxiety (GAD-7) in caregivers of DLB sufferers. This finding may explain why PA is related to increased care burden in DLB caregivers.

Many studies have reported that anxiety and depression during COVID-19 quarantine are increase, while sleep quality is the opposite (36–38). We also observed that the sleep quality of caregivers is associated with increased GAD-7, PHQ-9 and ZBI in AD caregivers, which is consistent with our previous study and many similar studies (39). However, why sleep quality in caregiver of DLB sufferers is not associated with an increasing care burden is due to the ever-heavy psychiatric symptoms at night which caregivers has used to, and this phenomenon is called psychological resistance. The sleep change of caregivers may arise from increased caring time at night and concern regarding the nighttime activity of their patients and stepped anxiety, which is also termed caregiver vigilance (40). The COVID-19 pandemic has had a significant impact on elderly individuals worldwide, particularly on those with cognition impairment and their caregivers family members. The COVID-19 pandemic recurred and worsened during late 2021, and a new mutation of the virus persisted (41). Many governments reinforced their quarantine policies, which represented a new challenge for patients and caregivers. Our study results indicate that caregivers should attach importance to sleep quality, attempt to maintain regular physical activity and social contact even during quarantine by means of increased home-based workouts (42), take specialized internet courses and training (43), and even seek support over the telephone (44).

The advantage of our study is that we conducted a longitudinal 1-year follow-up study after confinement during the COVID-19 pandemic. We used a variety of standardized scales to evaluate anxiety, depression and care burden at two different time points to discover similarities and differences between distinct dementia subtypes. Additionally, we quantified PA and social contact and tried to predict risk factors for worsened psychological status and caring burden. We also adopted caring time to reflect total disease burden instead of using MMSE, MoCA or NPS evaluation alone. This approach might have helped us avoid the difficulty in assessing the total relationship due to their interaction. A previous study suggested that women were more easily affected with anxiety and depression and thus bore more burden. We adjusted for gender when investigating the correlations among PA, social activity and anxiety, depression and care burden. We also investigated the impact of sleep quality on anxiety, depression and care burden caused by confinement, a focus rarely seen in caregiver studies. Nevertheless, our research has several limitations. For one thing, it was a single-center study with a small sample size. Therefore, the statistical analysis was limited, and the findings should be regarded with caution. In addition, memory bias is not negligible. A healthy control group under the COVID-19 pandemic and lockdown was not included in this study. Confinement and lockdown diminish PA and social contact while also introducing additional issues, such as a poor diet, which should be discussed in future research. Other related research on caregivers is currently underway.

## Conclusion

Caregivers of patients with AD, DLB and MCI exhibited different progressions of mental status, and care burden worsening during the COVID-19 pandemic. Caregivers of DLB sufferers showed rapid worsening after China's 12-month lockdown. Reduced PA and social contact and worsened sleep quality during confinement had a long-term impact on the mental status and care burden of caregivers. During quarantines and lockdowns, caregivers should maintain home-based exercise routines, engage in regular social contact and emphasize sleep quality.

## Data Availability Statement

The original contributions presented in the study are included in the article/supplementary material, further inquiries can be directed to the corresponding author/s.

## Ethics Statement

The study was designed and performed in accordance with the Declaration of Helsinki, and written informed approval was endorsed by all participants.

## Author Contributions

YJ and SL designed the study. XB and JX wrote the manuscript. QM contributed to the collection and verified of clinical data. XB performed the statistical analyses. JG, X-DW, and HW contributed to the interpretation and discussion of the results and reviewed the manuscript. All the authors and the collaborating author contributed to manuscript revision and read and approved the submitted version.

## Funding

This work was supported by the National Natural Science Foundation of China [grant number 82171182], Science and Technology Project of Tianjin Municipal Health Committee [grant number ZC20121 and KJ20048], and Tianjin Key Medical Discipline (Specialty) Construction Project.

## Conflict of Interest

The authors declare that the research was conducted in the absence of any commercial or financial relationships that could be construed as a potential conflict of interest.

## Publisher's Note

All claims expressed in this article are solely those of the authors and do not necessarily represent those of their affiliated organizations, or those of the publisher, the editors and the reviewers. Any product that may be evaluated in this article, or claim that may be made by its manufacturer, is not guaranteed or endorsed by the publisher.
